# A Word for Poor Women

**Published:** 1891-02-14

**Authors:** 


### The Hospital

A Weekly Journal of

### Science, Medicine, Nursing, and Philanthropy.

*For Hospitals, Asylums, Infirmaries, Dispensaries, Medical Practitioners, Students, Nurses, and the Charitable Public.*

Vol. IX.—No. 229.] [February 14, 1891.

### A Word for Poor Women.

In the stormy controversy which is now raging in the medical world about the Midwives' Registration Bill there is danger that the principal persons concerned, namely, poor lying-in women, may be forgotten. The majority of medical men, we feel sure, are anxious that all women shall have skilled attendance in confinement. Honest and right-minded doctors can no more help desiring to see women skilfully treated at such a crisis than the operating surgeon can rid himself of an instinctive impulse to catch up a bleeding artery with his forceps. A practitioner who, knowing the risks attendant on parturition, can be indifferent about the safety of the large number of women who do not and cannot employ doctors for their confinement, is a monster. Now medical men as a class are not monsters.

There are one or two persons who have caused a great bustle and stir about very small matters. Unfortunately, the class of practitioners who mostly attend midwifery are not what may be called men of the world. They have not time to be so. The life of a general practitioner in a poor town or neighbourhood is one of continuous slavery. He must not only work, and work hard, all day, but too often he must give at least a considerable portion of every third night also to his duties. A man who lives in this way has very few opportunities for increasing his knowledge of the world; nor has he much mental energy left after his day's work for the critical study of newspapers and books. Moreover, he feels the pressure of his circumstances very keenly; he is conscious of being hardly put to it to make both ends meet; and he is, therefore, always in a temper of mind that is ready to resent any attempt at abstraction, however small, from his hardly-earned gains. He thus falls an easy victim to the impulsive rhetorician, who, mounted on a fiery steed, proclaims a wolf on the near horizon.

The class of practitioners we have named, than whom there is no more meritorious class in the whole profession, are the persons whose interests are most largely concerned in the proposed Registration of Midwives. We ask them to dismiss from their minds all the rhetoric of partisans, and to decide the case in the court of their own judgment and conscience. They are much better able to decide for themselves than anybody else is able to decide for them: they have merely to consult their common sense and their sense of justice. If they will, by an effort of imagination, put themselves in the place of the poorest lying-in women, we are quite sure they will arrive at but one decision, and that decision will be the right one.

The really important facts about midwives' training and registration all lie in a nutshell; and these, which are not disputed, we will now state once more to our medical readers. The first is this: that at the present time 60 per cent. of poor women in large manufacturing towns are attended solely by midwives. Now, this 60 per cent. represents in the aggregate four hundred and fifty thousand women —nearly half a million. But it also represents four hundred and fifty thousand, or nearly half a million newly-born, and, therefore, most pitifully helpless babies; that is to say, nine hundred thousand distressed and helpless human beings have their lives placed at the mercy of midwives in this country alone every year. No law which the State can make will take these mothers and babies away from the charge of midwives and place them under the care of competent medical practitioners. The poverty of the poor women is such that it is with them a case of the help of the midwife or of no help at all.

We venture to make this assertion—that everybody who is informed of these facts ought at once to feel that since 60 per cent. of poor women are compelled to be content with midwives, they shall at least have midwives to attend them who possess reasonable knowledge and skill. Everybody, we say, ought to feel this; but the persons who ought to feel it and insist upon it most of all are the very medical practitioners who practice midwifery upon a large scale. And why? Because they are daily cognisant of the perils and dangers to which poor women are exposed, in their small homes and amid their miserable surroundings, at the period of childbirth. If the general public take little or no interest in the fate of the half-million of poor mothers who are annually attended by midwives, they may be partially excused on the ground that they knew little or nothing of the subject. But medical men know all about it; and they cannot excuse themselves, except on the condition of thereby proving that they are deficient in one of the commonest elements of humanity. We insist and urge upon every medical man's conscience, that it is his duty—a duty which it will be wicked to thrust aside—to consider this question as a man, to put away from him all the arguments that appeal merely to his selfishness; and to make up his mind that whatever other people may say and do, he will say and do that which is right and best for the women, whose helplessness is their only plea. If ever woman makes a pathetic appeal to man for help and defence it is in the hour of child-birth; and to what heart can her appeal be addressed if not to the generous instincts of those men who know by daily experience the sore straits and dangers which assail poor women as the consequence of their poverty alone? Medical men have not hearts of stone. They may for a time allow themselves to be drawn away from the real point at issue by appeals to selfishness and the cunning use of rhetoric. But when they are polled one by one—and each man owes it to himself to vote according to his conscience—there cannot be the slightest doubt that an overwhelming majority will boldly take the side of chivalry and the defence of those who will have no defenders at all if doctors desert them.

The second fact for practitioners to bear in mind is the excessive mortality among lying-in women who have no skilled assistance as compared with the mortality among those who are attended by trained midwives. Non-professional readers will be both surprised and shocked to learn that in England and Wales one mother in every two hundred dies in child-birth. But of those women who are attended by trained midwives belonging to lying-in hospitals, only one in every six hundred and fifty die. That is to say, there are more than three times as many deaths among the women who have not the means of paying for skilled attendance in their confinements as there are among those who can afford to pay the moderate fees of midwives. We take it for granted that medical men are quite familiar with these facts. But what an appalling state of things this is, when we come to consider it! For every one woman who dies in child-birth under skilled attendance, three die under unskilled: that is to say, no less than three thousand mothers die every year in England and Wales alone for want of the skilled attendance which competent midwives could give them.

Now, whoever is not ashamed of this truly terrible fact, the present writer is—and that both as a man, as a politician, and as a doctor. The thing is disgraceful, alike to the intelligence, the political capacity, and the humanity of the English nation. But, if it be disgraceful to the nation, most of all is it disgraceful to the medical men of the nation. For, just as the clerical profession charges itself with the religious interests of the people, and the judges and lawyers charge themselves with the maintenance and improvement of the law, so should the medical council, the colleges and hospitals, and the physicians, surgeons, and general practitioners of this country charge themselves with the care of the life and health of the people. We are aware that we may be met by the sneer that the people, neither directly by themselves nor indirectly by their Government, pay the medical profession for anything beyond the particular care which individual doctors give to individual patients. And what if that be even so? Do medical men owe no obligations to their humane and honourable calling? Is there a doctor anywhere who does not understand the meaning of *esprit de corps*? Or is the *esprit de corps* of physicians and surgeons confined to the extirpation of unqualified practitioners, and to insistance upon the minutest details of conventional professional etiquette? The greatest of all the obligations that devolve upon the medical profession are those which are owed to humanity; and those obligations that stand out prominently, even amongst the greatest, are the necessary services demanded by the sick, and most of all by those particular sick who are at one and the same time sick, poor, and at a perilous crisis of life. Undoubtedly it is the duty of the medical profession to insist upon the sufficient training and the registration of midwives, even though every other department of the State be indifferent or hostile.

